# Essential, not peripheral: Addressing health care workers’ mental health concerns during the COVID‐19 pandemic

**DOI:** 10.1002/1348-9585.12169

**Published:** 2020-10-02

**Authors:** Christi J. Guerrini, Eric A. Storch, Amy L. McGuire

**Affiliations:** ^1^ Baylor College of Medicine Houston TX USA

**Keywords:** COVID‐19, mental health, pandemic, psychological distress

## Abstract

In this Opinion, we synthesize recent evidence regarding the mental health impacts of the pandemic with an emphasis on health care workers. Departing from the literature that has already been published on this topic, we focus on health care workers with mental health concerns that preexisted the pandemic and discuss evidence suggesting that this population has suffered disproportionately from pandemic conditions.

As experience managing the COVID‐19 pandemic accumulates and cities across the world reopen, clinicians and public health officials are beginning to assess and remedy the damage that months of resource triage, economic torpor, and social distancing have had on patient health. The damage is expected to include complications resulting from postponed treatment of health concerns that emerged and acute health events that occurred during the shutdown. It also will likely include issues caused by poor management of chronic conditions prompted by pandemic‐related disruptions to diet, exercise, and drug regimens. Postponement of screening and other preventative health measures has almost certainly contributed to disease burden as well.

In addition to harms to physical health, it has become clear that the COVID‐19 pandemic has taken a serious toll on mental well‐being. Increased anxiety related to the pandemic has been found among the general population. In a US‐based poll fielded by the Kaiser Family Foundation in March 2020, almost half of respondents stated that worry or stress related to COVID‐19 has had a major (19%) or minor impact (26%) on their mental health.^1^ Similarly, the US National Center for Health Statistics and Census Bureau found that between April 23 and May 19, 2020, approximately 35% of respondents showed clinically significant anxiety or depressive symptoms.^2^ By comparison, when the same survey was fielded from January to June 2019, only 11% of respondents showed either symptom. For some, these experiences will result in new mental illness diagnoses or aggravate preexisting mental health concerns, including substance abuse disorders. The Well Being Trust and Robert Graham Center estimate that over the next ten years, the United States will experience tens of thousands of “deaths of despair,” or deaths due to drugs, alcohol, or suicide, that are attributable to the rise in unemployment, isolation, and uncertainty caused by the pandemic.^3^

Concerns have been raised about disproportionate mental health burdens on specific populations, including health care workers and first responders caring for infected patients, sometimes without adequate personal protective equipment. In China, studies have reported high levels of depression, anxiety, and insomnia among health care workers in direct contact with COVID‐19 patients and concluded they might require psychological support or intervention.^4^ Similarly, in a survey of almost 1,400 front‐line and second‐line health care workers in Italy, half reported posttraumatic stress symptoms and one quarter reported symptoms of depression.^5^ Health care workers in the United States have spent less time on the pandemic battlefield and so evidence of impact on their mental well‐being is still limited. However, narrative accounts of distress, fatigue, and burnout suggest that the burden on healthcare workers in the United States, as in other parts of the world seriously impacted by the virus, has been significant.^6^, ^7^, ^8^

Thus far, consideration of these mental health impacts has not distinguished between issues that arose before versus during the pandemic or has focused exclusively on new disease burden. Little is known specifically about consequences for individuals with mental health concerns that preexisted the pandemic. In the United States, this patient population comprises an estimated 18.1% (43.6 million) of adults.^9^ Notably, health care workers have consistently been found to experience higher rates of mental health issues than the general population even in non‐pandemic conditions.^10^ Depending on the disorder, care for persons with mental health concerns can be resource intensive and complex. Some illnesses, such as depression are treated as chronic or recurrent, with symptoms that are more or less debilitating, and more or less easy to manage, at different points in time.

As health care systems ramp back up their non‐emergency services, there is reason to believe that there will be a surge in demand for mental health services, especially among health care workers. From April 17 to 22, 2020, we conducted an online survey of 1,366 individuals located in the United States using Amazon's Mechanical Turk. Of the 1,123 respondents who had not participated in pre‐pandemic treatment with a mental health professional, 51% of those employed in health care systems, compared to 22% of other respondents, reported being very or somewhat likely to seek treatment with a mental health professional to help them address a pandemic‐related mental health issue (OR = 3.7; *P* < .0001). This difference was even more pronounced for respondents who had participated in pre‐pandemic mental health treatment. Of these 243 respondents, almost all (95%) of those who worked in health care systems, compared to 34% of those who did not, had increased or wanted to increase treatment frequency during the pandemic (OR = 40.0; *P* < .0001). Similarly, 82% of health workers, but only 33% of other respondents, were very or somewhat likely to increase treatment frequency post‐pandemic (OR = 9.4; *P* < .0001).

As bioethicists and hospital administrators debate whether institutions have an ethical obligation to prioritize medical care for health care workers,^11^ consideration should be given to whether that duty encompasses psychological support. Many institutions already are stepping up to provide these resources. Our own has amped‐up mental health resources for faculty, staff, and learners to include counseling sessions and support groups available via a telehealth platform and a self‐guided cognitive‐behavioral therapy app. Moving forward, we believe that these services should be provided with special attention to those with preexisting mental health concerns given that they appear to be suffering disproportionately from pandemic conditions. Identifying these individuals is complicated by the known reluctance of health care workers to seek psychological help,^10^ and there might also be privacy issues associated with targeting mental health communications to those having a record of such treatment. We believe the better approach is for institutions to cast a wide net and send all workers frequent reminders of the mental health resources that are available to them, promote a culture that encourages uptake of these resources, and ensure that they will be provided in strict confidence.

Given that there is no clear end in sight to the pandemic, we should expect many mental health services to continue to be offered remotely. Enthusiasm for telehealth during the pandemic has been high,^12^ and mental health professionals in particular have made tremendous efforts to implement in just a few months some telehealth programs that were originally planned to roll out over several years.^13^ However, these programs may be experienced as disruptions in care. Our survey respondents, for example, described a number of issues associated with remote mental health treatment, ranging from technical problems, to distractions from household members, to a perceived “weirdness” and “awkwardness” associated with visits via telephone or web conference. As online therapy sessions, support groups, and mental health apps proliferate, more data are needed to better understand factors associated with telehealth experiences, with a goal of making them more accessible to and effective for all patients. In the meantime, it should not be assumed that friends and family are able to fill gaps in emotional support during the pandemic, especially for those requiring professional treatment. Given that many health care workers are working increased hours in addition to physically distancing themselves from loved ones to prevent transmission of infection, they might be at heightened risk of escaping these supportive safety nets. Now more than ever, health care institutions must commit to ensuring that mental health resources are within easy reach of all who might benefit from them, but especially their own workers, during the pandemic and after it ends, whenever that day comes to pass.

## DISCLOSURES

*Approval of the research protocol*: The protocol for the survey described in this Opinion was approved by Baylor College of Medicine Institutional Review Board (Protocol H47626). *Informed consent*: Consent to participate was obtained by clicking on the forward arrow on the survey's first page. The requirement for written documentation of informed consent was waived because the risks to respondents were determined to be minimal and the study involved no procedures for which written consent is normally required outside of the research context. *Registry and the registration no. of the study/trial*: N/A. *Animal studies*: N/A. *Conflict of interest*: The authors have no competing interests to disclose.

## AUTHOR CONTRIBUTIONS

CJG led the drafting of the manuscript and data analysis. CJG, EAS and ALM contributed to the conception and design of the survey. EAS and ALM contributed to drafting the manuscript.
